# Supplementary material for: Are carbon emissions trading and green financial instruments synergistic? -Comprehensive quantitative research based on content analysis
Source: PLoS One. 2024 Mar 7;19(3):e0298601. doi: 10.1371/journal.pone.0298601 (PMC10919601; doi:10.1371/journal.pone.0298601)
Supplement: S1 Data — (ZIP) [file pone.0298601.s001.zip › Data/地级市Z_F工作报告.zip!/╣π╢1⁄2/╘╞╕í╩╨/└┤╫╘╣1⁄2╓┌║┼í╛┬φ┐╦╩2╛▌═°í┐_2018.docx]

各位代表：

我代表市政府向大会作政府工作报告，请予审议，并请政协委员和其他列席人员提出意见。

2017年工作回顾

2017年，是我市全面建成小康社会的关键之年，也是新一届政府工作的开局之年。在习近平新时代中国特色社会主义思想指导下，在中央、省委省政府和市委的正确领导下，在市人大及其常委会和市政协的监督支持下，市政府深入贯彻省委省政府促进粤东西北地区振兴发展和创新驱动发展战略，紧紧围绕建设现代生态城市目标，主动适应把握引领经济发展新常态，坚持生态立市、产业兴市、特色美市、改革活市、依法治市，狠抓“招商、建城、改革”，以推进供给侧结构性改革为主线，突出发展经济、保障民生两大重点，经济社会发展取得新成绩，较好地完成了市六届人大一次会议确定的目标任务。

2017年，全市经济发展稳中有进。预计全市实现地区生产总值863亿元，同比增长（下同）6%；规模以上工业增加值265亿元，增长5%；固定资产投资总额627亿元，增长6%；工业投资346亿元，增长12.5%；地方一般公共预算收入57.49亿元，增长0.11%；税收总收入102亿元，增长14.8%；社会消费品零售总额381.81亿元，增长10.6%; 外贸进出口总额133.24亿元，增长4.3%；城乡居民人均可支配收入 17839元，增长8%；12月末，金融机构本外币存款余额1120.86亿元、贷款余额731.45亿元，分别增长9.2%、12.3%。实体经济规模进一步壮大，规模以上工业企业发展到966家，比上年增加91家。经济结构继续优化，三次产业比重调整为18.9∶42.0∶39.1。

一、产业发展有新成效

新产业新业态蓬勃发展。全市共引进“四新一特^①^”项目81个，计划总投资241亿元。云计算及信息服务业提速发展。云计算数据中心一期加快建设，“云谷”道路及绿化景观工程全面完善，配套产业创业孵化基地、创新设计中心投入使用。“核高基^②^”技术攻关取得突破。先进装备制造业加快发展。成功纳入珠江西岸先进装备制造产业带。氢能产业发展水平处于全国同行前列。建成氢燃料电堆、飞驰新能源汽车两条生产线；建成氢能标准化创新研发中心、佛山（云浮）氢能产业与新材料发展研究院两个研发中心；建成氢能公交车、加氢站两个运营平台，在国内率先开通氢能公交车商业化示范运营，建成加氢站1座、在建5座。初步形成研发、孵化、生产的产业链，成为全国氢能产业基础最好、发展最快、集聚度最高的地区之一。生物医药产业集聚发展。全市已培育发展规模以上医药企业16家。我市获批建设广东省现代特色南药试验区。健康养生旅游产业提质发展。新兴县荣获“中国最具魅力文化旅游名县”和“中国最美乡村休闲旅游胜地”称号。六祖小镇成为广东特色小镇创建工作示范点。现代特色农业优化发展。全市农林牧渔总产值260.8亿元，增长4.2%。新增广东农业公园1个，全省休闲农业与乡村旅游示范镇2个、示范点3个。罗定稻米荣获“中国2017百强农产品区域公用品牌”和“2017最受消费者喜爱的中国农产品区域公用品牌”。电子商务快速发展。罗定市成为省级电子商务进农村综合示范地区。16家电商和快递企业入驻新富云电商快递产业园。

传统产业加快转型升级。全市完成工业技改投资120.2亿元，增长27.2%。新增技术改造项目备案90项，涉及总投资40.78亿元，引导88家规模以上企业开展技术改造，推动4家规模以上企业开展机器人应用。凌丰集团6条不锈钢制品自动化生产线全面投产，万事泰集团获评2017年省级工业设计中心。大力实施企业成长工程，获批全省促进中小微企业上规模工作示范市。推动企业上市融资，通力定造、惠沄钛业、新金山生物科技成功在新三板挂牌。组建国有独资的云石石材产业投资有限公司并开始运作。成功举办第十四届云浮国际石材科技展览会暨第八届云浮石文化节。港口物流业加快发展，保税仓储区加快建设，云浮新港全年吞吐货物总量突破22万标箱。

重点项目建设扎实推进。40个市管省重点项目完成投资84.59亿元，完成年度计划投资的102%；116项市重点项目完成投资197.14亿元，完成年度计划投资的100.67%。引进合同项目173个，其中投资超亿元的项目92个，超10亿元的项目14个。承接73个珠三角地区转移项目。大力实施佛山云浮产业共建行动计划，推动28个超亿元项目落地建设。“一区一龙头项目^③^”推进成效显著，引进铨欣智能照明、东鹏智能家居等投资额10亿元以上龙头项目10个，总投资159.8亿元。2017经贸活动共落实投资5000万元以上的“三个一批^④^”项目211个，总投资额881.75亿元。溢康通空气弹簧项目一期、中顺洁柔纸业二期等项目建成投产，广东甘美药品保健食品生产项目、衍生集团健康医药产业等项目动工建设，海尔云浮（罗定）创业园等项目进展顺利，华润西江发电厂等项目前期工作取得积极进展。

二、创新驱动激发新活力

高新技术企业加快培育。新增国鸿氢能科技、飞驰新能源汽车等高新技术企业16家，总量达到39家。新增广东省高新技术产品83个，增长245.8%。研发经费支出占GDP比重提高到0.8%，科技进步贡献率达54.5%。企业知识产权创造和运用能力不断增强，有效发明专利量、专利授权量分别增长23.52%、23.25%。云帕斯科技开发有限公司的云浮互联网远程医疗项目获第四届世界互联网大会最佳创新奖。

创新平台加快建设。新建省级、市级新型研发机构各1家。新建省级工程中心10家、市级工程中心8家、市级重点实验室3家、省级产业技术创新联盟2家和省级企业科技特派员工作站1家。云浮市农业生物工程创新基地、仲凯禅农（罗定）农业研究院、云浮循环经济工业创新研究院建设进展顺利。云浮创新设计中心成为广东省2017年众创空间试点单位和省级新型研发机构。

优秀人才加快集聚。落实“1+3”人才政策^⑤^，实施“千人计划^⑥^”等引才工程，引进各类人才2612人。全市新增副高以上高级职称专业技术人才327人，引进硕士研究生以上学历人才233人、基层医疗卫生特殊紧缺人才168人。全市首家省市共建“人才驿站”挂牌运作，成功举办广东省第九届“海外专家南粤行”云浮专场活动、海内外高层次人才云浮行活动。

三、发展环境形成新优势

交通基础设施日臻完善。完成交通基础设施投资66.2亿元，完成年度计划投资的100.9%。汕湛高速清云段、汕湛高速云湛段、罗信高速、怀阳高速二期、高恩高速等5个高速公路项目加快建设。建成新城快线南延段、西江(界首至肇庆)航道扩能升级工程（云浮段）等项目。东部快线、新城快线二期等项目加快建设。完成国道324线、省道368线等公路路面改造工程。

园区提质增效明显。全市园区累计投入基础设施建设资金112亿元；累计入园项目531个，总投资897.08亿元；实现规模以上工业增加值87亿元，增长8%。佛山（云浮）产业转移工业园引进项目17个，实现规模以上工业增加值75亿元；新规划建设的腰古片区完成征地5400亩。罗定片区引进项目37个，完成固定资产投资18亿元。佛山顺德（新兴新成）产业转移工业园引进项目141个，实现规模以上工业增加值14亿元。云安绿色日化产业基地引进项目 16 个，计划总投资20.9亿元。

城市扩容提质加快推进。中心城区“六大增量提质工程^⑦^”取得阶段性成果。云城片区、六都片区控规覆盖率达100%。云浮新区全年新动工建设项目22个，年度完成投资11.47亿元，续建项目13个，年度完成投资8.19亿元。市体育场、西江新城文化中心、地下综合管廊等项目加快推进。市中心城区主干道升级和绿化景观改造完成，育才路、乐谊路东段等道路贯通。英东体育馆地下人防工程稳步推进，蟠龙天湖景区升级改造工程投入使用，田心片区综合改造提升工程顺利推进。全市新建城市公园12个，改造升级公园15个。光纤入户率达56.85%，超出省下达目标任务15.85个百分点。城区管道燃气实现全覆盖。成功创建国家园林城市，国家卫生城市、省文明城市顺利通过复审。郁南县、新兴县获广东省县级文明城市提名，新兴县成功创建国家卫生县城。

四、新农村建设有新进展

新农村示范村建设成效初显。全市105条省定贫困村同步开展社会主义新农村示范村建设，实现卫生站全覆盖，“三清^⑧^”“三拆^⑨^”基本完成，“三整治^⑩^”工作扎实推进。101条行政村实现通快递物流。851条自然村完成通村委道路硬底化改造。罗定市、郁南县省级新农村连片示范建设工程通过省绩效评估。新兴县龙坪村被命名为省级文明村建设示范点。

农村人居环境不断改善。完成新农村公路建设160公里。农村生活垃圾有效处理率达95.01%，走在全省前列。63个镇（街）建成垃圾转运站，8788条自然村建成垃圾收集点。224条村生活污水处理设施建成投入使用。郁南县成为“全国农村生活污水处理示范县”。

精准扶贫、精准脱贫工作扎实推进。帮扶到村项目906个、到户项目27.18万个，全市有劳动力贫困户人均可支配收入7526元，处于全省中上水平，预脱贫人数达42685人。农村危房改造开工建设2200户，竣工2048户。

五、生态建设有新作为

污染防治工作成效明显。深入推进“大气、水、土壤污染防治行动计划”和“南粤水更清行动计划”。坚决按中央和省环保督察要求落实整改，2017年底新兴江、南山河水质达Ⅳ类标准，罗定环保基础设施建设基本完成。全面加强石材、水泥、硫化工、陶瓷等行业污染治理，市区空气质量优良率达91.8%。完成44个饮用水水源地标准化建设。建成迳尾水库备用水源项目。完成治理中小河流201公里。市城区污水处理率达90.8%，市中心城区截排系统完善及污水处理厂扩建项目全面动工。城市生活垃圾无害化处理率达100%。全面实施“河长制”，设置市、县、镇、村四级河长1043人。完成黑臭水体阶段性整治。单位GDP能耗下降3.8%。严格落实环保“三同时”制度，拒绝或劝退高污染项目超过20个。淘汰黄标车4297辆。

新生态建设进一步加强。完成森林碳汇造林3.5万亩，迹地更新造林19.06万亩，完善提升生态景观林带109.8公里，新建生态景观林带29.5公里，森林覆盖率达69.7%。新建森林公园9个、湿地公园2个、街头绿地3个。城市建成区绿地率达38.34%，绿化覆盖率达42.05%。荣获“2017中国绿色发展优秀城市”称号。

六、综合改革取得新突破

供给侧结构性改革成效明显。“三去一降一补^⑪^”取得阶段性成效，56家“僵尸企业”出清重组，取缔2家地条钢企业。商品房去库存面积70.54万平方米。“四平台五基金^⑫^”累计撬动信贷投放和直接融资10.3亿元。降低企业融资成本1343万元、用电成本1.15亿元。投入54.4亿元开展补短板工程。全面完成全市158个中心村新一轮农网改造升级任务。农业供给侧结构性改革深入推进，建成市、县、镇、村四级农产品质量安全监管网格体系，“国家生态种养农业标准化示范区”加快建设。

各项改革深入推进。“放管服”改革十大行动计划^⑬^扎实开展，行政审批时限比法定时限压缩70%以上，压减证明事项累计153项，累计清理规范行政审批中介服务259项。机关事业单位养老保险制度改革顺利推进。公证体制和人民监督员选任管理方式改革顺利完成。商事制度改革进一步深化，实施“多证合一”和企业简易注销改革，市场主体达91325户。国际贸易“单一窗口”标准版在我市推广运行，通关效率进一步提高。全面启动国有林场改革。市直政府采购和公共资源交易改革顺利推进，工程交易、土地与矿业权交易、产权交易等电子交易系统投入运行。全国农村改革试验区、国家农村产业融合发展试点等工作深入推进。农村土地承包经营权确权登记颁证加快推进。

七、社会事业取得新进步

全年民生类支出132.07亿元，占全市地方一般公共预算支出的72.37%。

教育卫生事业加快发展。广东药科大学云浮校区累计投入建设资金5.22亿元，项目一期主体建筑封顶。教育创现累计投入9.4亿元。39个镇通过省级强镇复评。公办义务教育学校全部建成标准化学校。普通高中全部达到市一级以上，实现优质高中全覆盖。继续实施“千人互派计划^⑭^”。市中医院管理移交广东药科大学并作为附属医院挂牌运营。罗定市罗镜镇、新兴县稔村镇、郁南县连滩镇中心卫生院改造升级为县级第二人民医院建设项目全面动工，24间镇卫生院标准化建设稳步推进，236间村卫生站公建规范化建设全面完成。深化医药卫生体制改革，全市所有公立医院全面取消药品加成。全面两孩政策落实到位，人口自然增长率8.07‰。计划生育管理服务水平进一步提高，罗定市、新兴县县级出生缺陷综合干预中心通过评审验收。

社会保障水平稳步提高。落实省、市十件民生实事资金53.27亿元。全面推进“大众创业、万众创新”，全市城镇新增就业2.1万人，扶持创业1286人，城镇登记失业率2.47%。城乡居民养老保险、城乡居民医疗保险参保实现全覆盖，企业职工基本养老保险省级统筹稳步实施，全面完成省下达的企业养老保险扩面任务。市福利院一期工程完成。全国部分省份农村特困人员供养服务机构社会化改革座谈会在我市召开。

文化体育事业不断进步。成功创建全国文明单位3个、全国文明村2条、全国未成年人思想道德建设工作先进单位1个。建立云浮市文化传承发展基地，完善16个镇（街）综合文化站设施，建成8个镇（街）文体广场示范点，市中心城区建成7个书吧示范点。255条行政村（社区）建成综合性文化服务中心。建成77个“扫黄打非”进基层示范点。我市“农村春晚”被列入全国文化共享工程“百姓大舞台”品牌项目。《禾楼舞》获中国文联最高奖项—“中国山花奖”；玉雕作品《藤牵硕果任君尝》首次入围山花奖。全民健身广泛开展，55个镇及所属行政村全部建成全民健身小广场。成功承办中国南粤古驿道文化之旅、南粤古驿道定向大赛等大型文化体育活动。

社会大局和谐稳定。平安云浮建设成绩显著。圆满完成党的十九大维稳安保特别防护期工作，实现了省委提出的“四个不发生^⑮^”，在党的十九大期间成为全省“零进京上访”和“零到省上访”的11个地市之一。全市63个镇（街）基本形成“网格+中心化+信息化”工作体系。率先在粤东西北地区建成综治和公共安全信息、视频监控系统。“全民禁毒”工程和全市治安查控网建设大力推进，建成全省第一个移动公安检查站。全市社区矫正中心全部建成，成为全国首个实现部、省、市、县、镇五级社区矫正指挥平台互联互通的地级市。安全生产和食品药品安全形势良好，全市产品质量监督抽查合格率连续三年稳步提升。认真贯彻军民融合发展战略，国防动员和后备力量建设得到加强。率先在全省建立市、县、镇、村四级复退军人服务平台。

八、政府建设有新成果

依法行政水平进一步提高。自觉接受人大法律监督和政协民主监督，认真听取民主党派、工商联、无党派人士及工会、共青团、妇联等人民团体意见建议，办理人大代表建议132件、政协提案149件，办结率100%。办理行政复议案件62宗。“七五”普法规划全面实施，市、县两级法治文化主题公园全面建成，法治建设“四级同创”活动全面推开。全国首部规范农村生活垃圾治理的地方性法规《云浮市农村生活垃圾管理条例》正式实施。

政府作风建设取得实效。政府系统“两学一做”学习教育常态化制度化拓展深化、引向深入。廉政建设责任制严格落实，行政监察和审计监督进一步强化。认真落实中央“八项”规定精神，“四风”整治不断深化，“三公”经费和会议费支出连续五年实现“零增长”，政风持续好转。

与此同时，民宗、外事侨务、港澳台、人防、统计、地方志、粮食、消防、档案、保密、科协、侨联、文联、残联、社科联、贸促、慈善、老区、老龄以及驻市外机构等工作取得新成绩。

过去一年，市政府深入学习习近平总书记系列重要讲话精神，把党中央的要求落实到政府具体工作中。党的十九大召开以来，市政府认真组织学习宣传贯彻党的十九大精神，切实把思想和行动统一到习近平新时代中国特色社会主义思想上来。聚焦落实党的十九大提出的新目标、新要求、新思路、新举措，按照中央、省委部署和市委要求，把党的十九大理论成果、实践成果和制度成果贯彻落实到政府工作中。针对云浮发展不平衡不充分的突出矛盾，开展产业、教育、医疗卫生、城市、创新能力、乡村振兴等专题调研，谋划高质量发展，加快现代生态城市建设。

各位代表！过去一年的成绩来之不易。这是省委、省政府和市委正确领导、科学决策的结果，是全市人民齐心协力、奋力拼搏的结果。在此，我代表市人民政府，向全市人民，向人大代表、政协委员，向所有关心支持云浮的同志们、朋友们，致以崇高敬意和衷心感谢!

在总结成绩的同时，我们必须充分认识当前发展存在的问题和不足：一是发展不平衡不充分仍是最突出的矛盾，实现与全省同步全面建成小康社会目标压力较大；二是产业集聚水平低，大企业、大项目较少，部分企业效益不佳，一些重点项目建设推进缓慢，土地、资金等要素制约仍然突出；三是环保突出问题还未得到彻底解决，大气、水、土壤污染防治任务还很艰巨；四是民生事业短板较多，基本公共服务供给能力与人民群众的需求还有较大差距；五是政府自身建设需要进一步加强，一些干部抓落实的主动性不够，办事效率有待提高等还一定程度存在。对于这些问题，我们必须高度重视，采取有力措施切实加以解决。

2018年工作安排

中国特色社会主义进入了新时代，我国经济发展也进入了新时代，社会主要矛盾已经转化为人民日益增长的美好生活需要和不平衡不充分的发展之间的矛盾，经济已由高速增长阶段转向高质量发展阶段。站在新的历史起点上，一定要保持正确的前行方向。

——我们要坚持以习近平新时代中国特色社会主义思想引领云浮发展。坚持党对经济工作的集中统一领导，不折不扣贯彻落实中央、省和市委的决策部署。坚定贯彻新发展理念，坚持稳中求进，正确处理稳和进的关系，努力实现更高质量、更有效率、更加公平、更可持续的发展。

——我们要永远把人民对美好生活的向往作为奋斗目标。坚持以人民为中心的发展思想，坚定践行根本宗旨，带领全市人民通过不懈奋斗创造美好生活。深入推进供给侧结构性改革，以高水平供给满足云浮人民升级变化的需求。

——我们要坚定不移走高质量发展之路。坚持以创新发展为动力，坚持节约集约用地，坚持环境友好型发展，把着力点放在实体经济上，不断转换发展动能，通过质量变革、效率变革、动力变革，推动我市经济高质量高水平发展。

——我们要大力实施乡村振兴战略。坚持农业农村优先发展，大力补齐“三农”短板，推动农村产业兴旺、生态宜居、乡风文明、治理有效、生活富裕，加快推进农业农村现代化。

**2018年市政府工作的总体要求是：**全面贯彻落实党的十九大和中央经济工作会议精神，以习近平新时代中国特色社会主义思想为指导，按照习近平总书记对广东工作重要指示批示要求，坚持稳中求进工作总基调，坚持新发展理念，紧扣社会主要矛盾变化，贯彻高质量发展要求，统筹推进“五位一体”总体布局和协调推进“四个全面”战略布局，紧扣省第十二次党代会和省委十二届二次、三次全会部署，以及市第六次党代会“一同步六提升”目标和市委六届三次全会精神，坚持“生态立市、产业兴市、特色美市、改革活市、依法治市”，坚决打好防范化解重大风险、精准脱贫、污染防治“三大攻坚战”，聚力实施乡村振兴战略，继续抓好“招商、建城、改革”，加快云浮振兴发展、全面建设现代生态城市、决胜全面建成小康社会。

**今年全市经济社会发展的主要预期目标是：**地区生产总值增长7%左右，规模以上工业增加值增长6%左右，固定资产投资总额增长8.5%左右，地方一般公共预算收入增长6%，社会消费品零售总额增长8%左右，进出口总额实现正增长，实际利用外资增长6%，居民消费价格涨幅控制在3%以内，城乡居民人均可支配收入增长和经济增长基本同步，完成省下达的节能减排任务。围绕上述目标，重点抓好八方面工作：

一、积极构建现代产业体系，提升经济发展质量

现代产业体系是现代化经济体系的重要组成部分。要加快发展新兴产业，促进传统产业转型升级，构建具有云浮特色的现代产业体系。

发展壮大新兴产业。大力发展“四新”产业。加快云计算及信息服务业集聚。积极构建云计算产业、三维大数据产业、国产自主可控产业鼎足发展的云计算大数据产业发展模式。以华为公司为龙头，依托云计算数据中心，推动各类基础云计算服务产业发展。成立云浮市三维大数据中心，推动三维技术、大尺度光场大数据等技术落地应用。深化“核高基”重大专项10-1课题应用示范，推动龙芯中科公司上下游企业落户，打造国产自主可控产业园。加快发展先进装备制造业。提速发展氢能产业，保持先发优势。推动国鸿、飞驰等项目扩产。推进与东风汽车、中国石化、中国石油、南方电网、山西美锦集团、深圳西湖集团深度合作，加快推进氢能商用车推广应用，加快高性能氢能电堆和氢能乘用车研发，建成加氢站6座。推动氢能小镇建设，打造完善的特色产业生态圈。推动现代农牧装备、环保装备、不锈钢自动化机械、石材机械、汽车零部件等制造业加快发展。加快发展生物医药产业。加快市健康医药产业园和罗定中药提取基地项目建设，培育10家以上生物医药龙头企业。加快发展健康养生旅游产业。促进旅游与文化、体育、农林、养生等产业融合，大力发展乡村休闲旅游。加快六祖故里旅游度假区创建国家5A级旅游景区，推动云城区、新兴县创建广东省全域旅游示范区。推动互联网、大数据、人工智能与实体经济加快融合，培育新增长点、形成新动能。

加快传统产业转型升级。继续实施企业成长工程，新增规模以上工业企业50家以上、限额以上商贸企业50家以上。大力支持企业开展技术改造，实现工业投资增长10%，工业技改投资增长15%。大力推动生产自动化和产品智能化，创建2个以上省级智能制造试点示范项目。规范石材企业生产经营管理。加强石材大师工作室建设。引导和鼓励企业特别是石材、不锈钢制品等传统产业进行股份制改造，推动2家以上企业登陆新三板。深入实施质量强市战略，推动新兴县创建广东省质量强县示范城市和全国不锈钢餐厨具产业知名品牌创建示范区。加快全国石材产业知名品牌创建示范区建设。

二、强化优质项目支撑，做大做强实体经济

优质项目是构建现代产业体系的重要支撑。要通过大力引进和建设优质项目，夯实发展后劲。

大力引进优质项目。加快引进一批财税贡献大、科技含量高、生态环保、节约集约用地、产业带动力强的优质项目，努力实现全年招商400亿元的目标任务。加强与珠三角产业共建和佛山市对口帮扶合作，年内引进珠三角60个和佛山15个优质项目。

推动项目入园发展。加快“两园六区^⑯^”道路、供水、供电等基础设施建设。支持有条件的园区稳步推进扩容。加快开发佛山（云浮）产业转移工业园腰古片区，力争年内动工建设5个亿元以上项目。力争园区工业产值超500亿元，规模以上工业增加值超110亿元。

狠抓重点项目建设。今年全市计划安排重点项目142项，计划总投资1341.4亿元，年度计划投资230.07亿元。年内建成广东药科大学云浮校区一期、云计算数据中心项目一期、市体育场等23个项目。推进华润西江发电厂全面动工建设。加快推进天然气主干管道云浮支干线项目（肇庆—云浮）前期工作。

三、坚持创新驱动，不断增强发展动力

创新是引领发展的第一动力，是建设现代产业体系的战略支撑。

加快培育高新技术企业。完善企业创新创业、金融服务、人才引进、知识产权制度等政策措施，大力提升企业自主创新能力。年内新增高新技术企业6家以上，省高新技术企业培育入库企业8家以上。大力推进企业研发机构建设，规模以上工业企业建立研发机构覆盖率达到20%，培育一批工程技术研究中心。

加快创新平台建设。加大佛山（云浮）氢能产业与新材料发展研究院、氢能标准化创新研发中心建设力度。加快建设广东药科大学云浮研究院和广药集团国家中药重点实验室云浮分部。创建国家技术标准创新基地（氢能）。鼓励石材、硫化工等企业与广东工业大学创新设计中心、中科院深圳先进技术研究院、云浮循环经济工业园协同创新发展研究院等加强合作。

大力引进培养优秀人才。用足用活“1+3”人才政策。继续实施引进高层次及基层医疗卫生特殊紧缺人才“千人计划”，年内引进60名硕士研究生以上学历的高层次专业人才、200名本科以上学历的临床医学类专业人才。以氢能源、生物医药、云计算大数据等现代产业为主的学科带头人、科技领军人才达到30人。

四、大力实施乡村振兴战略，提高农业农村发展水平

实施乡村振兴战略，是中国特色社会主义进入新时代抓好“三农”工作的总抓手，必须立足市情，以更有力的措施推动农业升级、农村进步、农民发展。

推动乡村产业兴旺。深入推进农业供给侧结构性改革，深化农村综合改革。坚持质量兴农、绿色兴农，发展壮大现代特色农业。巩固国家农产品质量安全市创建成果，农产品质量合格率保持在98%以上。按照“四区两带^⑰^”农业发展新格局，深入实施“五三一”现代农业发展战略^⑱^，抓好“米袋子”、“菜篮子”，做强现代畜牧业、稻米、南药、花卉、水果等传统优势特色产业，提升农业品牌。加快广东省现代特色南药试验区建设，争创国家级现代农业产业园（南药）。全面完成农村土地承包经营权确权登记颁证工作。实施耕地质量保护与提升行动，发展“智慧农业”，保障粮食安全。创新政府购买农业公益性服务机制，发展农业新业态，促进农村一二三产业融合发展。推进氢农业应用试验工作。实施新型农业经营主体和职业农民培育工程。继续抓好全国罗定绿色稻米产业知名品牌创建示范区、国家地理标志产品保护示范区（广东罗定）、新兴县国家农村产业融合发展试点示范县建设。推进郁南县省级农村集体产权制度改革试点。

推动乡村生态宜居。大力开展农村人居环境综合整治，继续推进105条省定贫困村新农村示范村建设，年内完成20户以上自然村“三整治”工作。结合氢能物流车推广应用，全力抓好行政村通物流工作。加大规划建设和管理力度，建好管好保护好运营好农村公路。大力开展镇容镇貌整治，继续推进农村改水改厕工作。加大农村污水处理力度，完成37个建制镇生活污水处理设施建设。全面完成村村通自来水工程。深入实施《云浮市农村生活垃圾管理条例》，全面实现农村生活垃圾无害化处理。广泛开展文明镇街、文明村等基层创建活动。

五、全力打好三大攻坚战，夯实全面建成小康社会基础

打好防范化解重大风险攻坚战。重点是防控金融风险，增强服务实体经济的能力。推进金融机构和产品去杠杆工作，银行业金融机构不良贷款占比与全省平均水平基本持平。合理控制银行融资项目、证券投资业务和保险资金投资业务杠杆水平，金融业务主要风险指标达到监管要求，确保不发生系统性、区域性金融风险。全面完成全市农合机构改制组建农商行。创新性引入保险资金支持支农支小领域，深化“信用云浮”建设，推动互联网金融创新发展。

打好精准脱贫攻坚战。深入推进“脱贫攻坚八项工程^⑲^”，突出抓好产业扶贫、就业扶贫、教育扶贫、医疗扶贫，落实义务教育、基本医疗、安全住房等保障政策，激发贫困人口内生动力。积极打造产业扶贫亮点，努力形成“镇有产业、村有基地、户有项目”的产业扶贫新格局。加强扶贫资金使用管理，提高财政帮扶资金使用效能。完善扶贫大数据信息平台建设。推进农村危房改造，大力改善贫困户居住条件。重点推进分散贫困人口脱贫工作，完成3.48万相对贫困人口年度脱贫任务。

打好污染防治攻坚战。推进林业重点生态工程建设，森林覆盖率达69.8%以上。单位GDP建设用地使用面积降至65公顷/亿元以下。全力抓好大气污染防治，重点推进PM_2.5_、PM_10_、粉尘和臭氧治理，确保空气优良天数稳中有升。全面落实“河长制”，治理中小河流88公里。实施分段治水工程，完成新兴江、南山河等河流污染整治工作。落实最严格的水资源管理制度，完成一级饮用水源保护区违法项目清理。完成云城区安塘街道、罗定市泗纶镇和罗镜镇污水处理厂提标改造，加快完善市中心城区和云城区河口街道、云安区富林镇、新兴县六祖镇等污水处理厂配套管网。完成云城区、罗定市、新兴县和郁南县4座生活垃圾填埋场续建工程。推进土壤污染防治，抓好畜禽养殖和农业面源污染防治。

六、全面推进交通和城市建设，提升综合竞争力

完善综合交通体系。全市交通基础设施建设计划投资51 亿元。完成汕湛高速云湛段建设和广梧高速思劳立交东移。加快汕湛高速清云段、罗信高速、怀阳高速、高恩高速等项目建设。积极推进罗定至德庆高速公路北段、广湛高铁等项目前期工作。谋划推动三茂铁路电气化改造等重大项目。推动云浮新港升级为全省“海上丝绸之路”重要支点。升级改造国省道98公里。全面开工建设国道234线南江口至双东高速出入口段。努力推动罗岑铁路复工建设和罗定机场扩建改建工程。扎实推进新城快线二期、东部快线、西部快线等城市组团交通建设，加快世纪大道等市区主干道快速化改造。加快交通、旅游、产业园区的网络对接规划。积极推进县乡公路升级改造和行政村道拓宽，新改建农村公路100公里。加快客运站升级改造和城乡客运发展。

推进城市扩容提质。坚定不移把云浮新区建设成为未来的中心城区。加快广盛路西延段、康盛路西延段、广东药科大学云浮校区周边道路等基础设施建设。抓好光明中小学、市体育场、市档案馆、市图书馆、新区水厂等项目建设，不断完善教育文化、医疗卫生等城市功能配套。推进中心城区城市道路、环卫设施、公共停车场等设施建设。推进华丰路、富民路北段、东方路等道路贯通工作。升级改造市城区育华路、龙华路等12条道路。升级改造广场、公园、步行道、慢行系统等公共设施，推进南山河两岸景观改造。推进“三旧”改造项目建设。完成田心片区综合改造，促进新老城区融合发展。巩固国家卫生城市、国家园林城市创建成果，全力创建全国文明城市、国家森林城市。大力推进“厕所革命”。抓好“海绵城市”建设试点，加快建设城市综合管廊5.3公里。推进信息基础设施建设三年行动计划，加快超高速无线局域网等级无线宽带网络建设，光纤入户率达到70%以上。加快罗定、新兴、郁南三个副中心扩容提质，推进罗定市粤桂边工业新城、新兴县禅意生态名城、郁南县绿色崛起示范县建设。

七、提升保障和改善民生水平，增强人民获得感和幸福感

优先发展教育事业。全力加快广东药科大学云浮校区建设，确保今年秋季如期招生。大力支持广州华立科技职业学院云浮校区升本工作。积极推进罗定职业技术学院扩容提质。全力推进教育现代化，确保市、县两级成功创建成为广东省推进教育现代化先进市、先进县（市、区）。加快推动市高级技工学校申报技师学院。积极推行远程教育，办好云浮开放大学。确保新兴县成功创建第四批国家级农村职业教育和成人教育示范县。努力扩大优质教育资源覆盖面，抓好市中心城区义务教育学校合理布局和均衡发展。确保恒大中学实现秋季招生。完善职业教育和培训体系，深化产教融合、校企合作。开展教育综合改革，深入实施强师工程，加大名校长名教师名班主任培养。推进中小学教师“县管校聘^⑳^”管理改革。全面落实学前教育到高等教育学生资助政策。

全力提升医疗卫生服务水平。推动健康云浮建设。实施市区域医疗中心建设规划，推进医疗联合体建设工作，加快市120指挥中心建设。建成市妇幼保健计划生育服务中心。抓好县级人民医院、中医院等升级工程，确保罗定市、新兴县、郁南县第二人民医院2019年建成投入使用。抓好镇卫生院标准化建设收尾工作，完成302间村卫生站公建规范化建设。加强中医名院、名科、名医建设，推广使用中医药适宜技术。推动民营医院发展。创新计生管理方式，落实全面两孩政策，强化计划生育目标管理责任制考核。抓好疾病防控和卫生应急工作。

提升就业和社会保障水平。大力实施就业优先战略，年度新增就业2万人，帮扶1000名就业困难人员实现再就业，城镇登记失业率控制在4%以内。持续优化“双创”环境，建设“好易工”线上公共服务平台、智能化就业创业服务大厅、产业共建高技能实训基地等综合服务平台。加快市福利院二期建设。全面推进以病种分值付费为主的多元复合式医保支付方式改革。加强对困难群体的兜底性保障。健全工资正常增长机制，加强劳动监察仲裁，畅通劳资纠纷法治化渠道。健全社会救助体系，提升城乡社区居家养老和残疾人康复服务能力。加快保障性安居工程建设和棚户区住房改造。

加快发展文化体育事业。大力完善公共文化服务体系，确保行政村（社区）综合性文化服务中心基本实现全覆盖。加快补齐文化产业短板，健全现代文化产业体系和市场体系。积极推进优秀传统文化传承发展“1234”工程，挖掘红色文化基因和地方特色文化内涵。宣传和利用好云浮十大名片，打造云浮文化高地。抓好文化惠民工程，活跃粤剧进农村进学校、农村公益电影放映、周末广场喜乐会等文化活动。推进广播电视村村通向户户通升级。推进文化市场综合执法改革，全面铺开“扫黄打非”进基层工作。加快体育场馆规划建设，建设一批社区体育公园和健身绿道。精心做好省第十五届运动会组团和备战工作，办好市第五届“市长杯”足球联赛、第十九届体育节、全民健身日等品牌健身活动。

加强和创新社会治理。围绕把云浮建设成为全省最安全稳定、最公平正义、法治环境最好的地区，全面深化平安云浮建设。创新基层治理，推进镇（街）人民调解委员会聘请专业人员专职从事人民调解工作。加快基层司法所规范化建设和公共法律服务中心建设。落实国家机关“谁执法谁普法”普法责任制。建设青少年法治教育实践基地。抓好综治信访维稳，大力推进“阳光信访、责任信访、法治信访”建设，有效排查化解社会矛盾。深化“中心+网格化+信息化”建设，积极推动“互联网+”社会治理，加快推进网信机构队伍组建，构建网络综合治理体系。依法打击“两抢一盗”、“黄赌毒黑拐骗”等违法犯罪活动，构建立体化社会治安防控体系。严格落实安全生产责任，预防和遏制重特大安全事故发生。切实做好森林防火工作。深入开展打击制售假冒伪劣商品行动，强化食品药品智慧监管和网格化监管。加快云浮市中药检测平台建设，推动郁南县创建省级食品安全示范县。扎实抓好民兵调整改革，深入推进军民融合发展，进一步巩固国防动员和后备力量建设。

八、加强政府自身建设，提升政府队伍能力水平

深入推进依法行政。坚决执行市人大及其常委会的决议和决定，自觉接受市人大法律监督、市政协民主监督，接受社会和舆论监督。办理好人大代表建议和政协委员提案。落实重大行政决策合法性审查制度。加强审计、统计等监督工作，扎实做好第四次全国经济普查。深化司法体制改革，完善法律服务体系，全面落实司法责任制。

大力提升政府效能。发挥市场在资源配置中的决定性作用和发挥好政府作用，确保市场竞争公平有序。继续深入实施“放管服”改革十大行动计划，进一步精简行政许可事项，全面实施行政许可标准化，深化“减证便民”行动。加快推动在线应用、行政审批基础信息共享以及审批实时在线监察体系建设。建立健全服务信息公开机制、办事督查机制和投诉举报机制，扎实推进“数字政府”改革建设。完善工作落实和问责机制，加强政府效能考核。加强财政预算管理，推行预算绩效目标管理。加强税收征管，调整和优化财政支出结构。推进事业单位改革和林权改革。

加强廉洁政府建设。坚持不懈推进政府系统全面从严治党和廉政建设，严明政治纪律和政治规矩。落实中央“八项”规定精神，深入整治“四风”和“为官不为”，严控“三公”经费支出。坚持以零容忍态度惩治腐败，构建亲清政商关系。加大力度惩治农村、社区、基层服务、扶贫等重点民生领域的不正之风和腐败行为。扎实开展“不忘初心、牢记使命”主题教育。

强化作风建设抓落实。各级政府领导干部要对标新任务、新要求，切实增强本领恐慌感和能力危机感，自觉在学习和实践中提高改革创新发展的本领能力，在抓落实中快行动，出实招，做成事。

各位代表，幸福都是奋斗出来的！新时代带来新机遇，呼唤新作为，让我们紧密团结在以习近平同志为核心的党中央周围，在省委、省政府和市委的坚强领导下，锐意进取，实干苦干，为推动云浮振兴发展、加快建设现代生态城市、决胜全面建成小康社会而努力奋斗！
